# The Development of an Interventional Package on "Receptive Vocabulary” For Cochlear Implanted Children

**Published:** 2019

**Authors:** Leila MONSHIZADEH, Roshanak VAMEGHI, Firoozeh SAJEDI, Fariba YADEGARI, Mehdi RAHIMI, Seyed Basir HASHEMI

**Affiliations:** 1 Pediatric Neurorehabilitation Research Center, University of Social Welfare and Rehabilitation Sciences, Tehran-Iran; 2 Speech and Language Department, University of Social Welfare and Rehabilitation Sciences, ehran, Iran; 3 Educational Psychology, Yazd University, Yazd, Iran; 4 Otolaryngology Research Center, Shiraz University of Medical Sciences, Shiraz, Iran

**Keywords:** Language disorder, Cochlear implant, Vocabulary development, Protocol, Educational package, Children, Receptive language

## Abstract

**Objectives:**

Considering the shortage of language intervention protocols which specifically concentrate on cochlear implanted children and considering the importance of timely language intervention in this group of children, the aim of the present study was to develop an interventional package on “receptive vocabulary” for cochlear implanted children.

**Materials & Methods:**

By reviewing the literature related to language acquisition theories in normal and language disordered children, as well as literature on production of intervention protocols, especially those for language impaired children, and also considering the normal process of language and speech development in normal children, the first draft of the intervention protocol was prepared. Then, the face and content validity of the intervention protocol was assessed by a Delphi team through three rounds and finally approved.

**Results:**

A language intervention protocol was developed to enhance receptive vocabulary in 12-48 months-old cochlear implanted children, based on cognitive, behavioral and developmental theories. This protocol includes 5 interventional stages: 1-Drilling and Imitation; 2-Modeling; 3-Motor training; 4-Deliberate error correction; 5- Reinstatement and Generalization. Each stage consists of the description of the aims of that stage, a list of techniques, the tools required, the detailed step by step explanation of the intervention, how re-enforcement must take place, and finally the indicators of success which permit to move forward to the next stage.

**Conclusion:**

The interventional package produced is believed to facilitate language acquisition in cochlear implanted children, according to expert qualitative assessment and approval. Experimental research is required for verification of this assumption.

## Introduction

Hearing loss is known as one of the most common etiologies of communication disorders (1). About 0.1% of live births suffer from profound congenital hearing loss. Asia is known as a continent with the largest proportion of hearing deficiencies, with 2.6 hearing impaired children born in every 1000 live births during a year. Annually 4000 hearing impaired newborn infants are born in Iran; yet, no absolute statistics exist in this regard (2, 3).

Hearing loss causes the child not to communicate with others simply. It is mostly because of the deaf child’s disability to acquire language in a natural trend that a normal child does during the first 2 yr of life (4-6). Many years ago the only way for deaf children to access sound, was to use hearing aids, while it could not provide severely to profoundly, and profoundly hearing-impaired children with different degrees of auditory stimuli (7). These days cochlear implantation is used as one of the best substitutions for hearing aids in order to help deaf children improve their auditory perception and speech and language acquisition (7, 8). Although cochlear implantation facilitates language acquisition especially for children received the device in the critical period of language development, enhancing and promoting language development in cochlear implanted children is highly in need of a systematic language intervention protocol (8). 

The history of designing language intervention protocols for language impaired individuals goes back to the 1960s and 1970s. The American speech and hearing association (ASHA) solely has performed more than 100 studies in this regard and has established various language intervention models by studying on autistic children, specific language impaired children and other language disordered groups (9). 

 Nowadays different language intervention protocols have been developed all over the world. These protocols commonly focus on the necessity of timely language intervention for language impaired children or those at risk of it. However, they mainly differ in terms of their focus on various aspects of language include syntax, semantic and pragmatic (10, 11). For example, one of the biggest companies of cochlear implantation in Austria, the Med-El company, is committed to developing rehabilitation services in the form of booklets. These booklets contain guidelines for different target groups of pediatrics, teens, and adults. The pediatrics guideline entitled “Little Ear” is a comprehensive guideline designed to help professionals and parents or other caregivers of young hearing aided or cochlear implanted children with information on preverbal communication, speech, language and auditory development. It is accompanied by helpful suggestions for parents to assist them in the facilitation of their young child’s communication skill development and consideration for school entry requirements (12).

The most commonly used intervention programs for all language impaired children in Iran, including children in cochlear implantation centers, are auditory-oral and auditory-verbal therapy. These two approaches are mainly concerned with improving spoken language by appropriate amplification technology to achieve maximum benefits of learning, a desirable auditory learning environment to facilitate the process of spoken language acquisition, mainstreaming of cochlear implanted children into systematic educational classes with suitable support services and finally encouraging parents for active participation in rehabilitation programs. In spite of their similarities, they differ mainly in that the auditory-oral approach encourages the use of lip-reading and facial expressions, while the auditory-verbal approach is not concerned with visual cues (13, 14). 

 Although each of these different intervention programs has its own positive effects for different target groups, to our knowledge, up to now no study has specifically proposed an educational package to facilitate language acquisition, especially regarding receptive vocabulary development in hearing-impaired children undergone cochlear implantation. Regarding the limited golden period remaining for language acquisition in most cochlear implanted children (4), as well as the shortage of language intervention protocols for this group of children, and the limited effect of cochlear implantation on language acquisition without a language intervention procedure (15), and also based on the importance of receptive vocabulary development in children and its noticeable correlation with language development generally (16), the main aim of this study was to design an interventional package for enhancing the receptive vocabulary development of 12-48 months-old Persian-speaking cochlear implanted children. 

Regarding the age range that was selected for which to produce this interventional package, in spite of the fact that cochlear implantation be performed in the first months of life, some challenges such as children’s associated disorders that must be cured before surgery, rather high expenses and long waiting lists for cochlear implantation have resulted in an actual increase of cochlear implantation age range to after 12 months of age in many cochlear implantation centers in Iran. Therefore this protocol was produced for this target group. 

The main aim of this article was to describe the process of content development of this package, which can serve as a guide for development of similar protocols for cochlear implanted children in other societies that are similarly short of such interventional material, as well as for development of intervention protocols for other aspects of language development in Persian-speaking cochlear implanted children. The protocol itself will be accessible on demand, for treatment of Persian-speaking children all over the country, as well as in Afghanistan, Tajikistan and elsewhere, possibly after some cultural, linguistic and alphabetical adaptation in countries other than Iran. 

## Materials & Methods

This descriptive study was performed in the University of Social Welfare and Rehabilitation Sciences, Tehran, Iran in two phases of review and qualitative phases.

In the review phase, described in detail before (17), literature relevant to various aspects of language development, including phonology, syntax, semantics and pragmatics, as well as language acquisition theories in normal and language disordered children, and different intervention programs for facilitating language development in language impaired children overall, and cochlear implanted children as one of the largest group of children with language impairments (5, 18-26), were reviewed. In order to achieve this goal, a team including 2 pediatricians and 2 speech and language pathologists began to search some relevant books as well as the Medline, Cochrane Library, Google Scholar, ISI web of science and Scopus databases, using the following keywords: Language disorder, Cochlear implant, Vocabulary development, Protocol, Educational package, Children, Receptive language. First, titles and abstracts of articles were screened, from which full texts of the most relevant ones were selected. Articles written in languages other than English or Persian were excluded. Studies accessed only in abstract form were also excluded. Finally, among 25 articles that were selected, only 11 met the inclusion criteria and were included in the study. According to the inclusion criteria, all review articles, expert opinion studies, non-experimental and experimental studies that clearly focused on behavioral and cognitive factors affecting language acquisition in children were selected.

Some points became the object of special attention in designing the protocol. A number of things must be considered and emphasized upon, in designing the protocol. For example, the cognitive abilities of the language impaired child must be enhanced by improving his semantic memory, alongside efforts to improve his receptive vocabulary (18, 24, 25). Moreover, the authors chose to focus on the child’s motor development, as well as emphasizing on his correct language behaviors, according to findings of some studies regarding correlation between movements and thought. A child’s movement ability promotes his investigation of the surrounding environment which may, in turn, facilitate his learning process (17, 27, 28). In addition, motor development prompts cognitive development and there is a neuronal connection between systems for action and language perception (27, 28). Thus, including motor movement training in a language intervention program might facilitate the process of language acquisition by enabling the child to manipulate objects and experience movements.

The authors were also determined to take advantage of specifically planned reinforcements from the therapist and parents especially when accompanied by their behavioral responses such as smiling or hugging, in order to facilitate the process of language acquisition in the child (25, 29, 30). 

Based on literature review and several group discussions held by the research team the first draft of the protocol was prepared. Thereafter, the qualitative phase was begun. In order to carry out this phase, a group of 10 expert pediatricians, speech and language pathologists and experts in linguistics as members of a Delphi team, was organized for face and content validity analysis of the first protocol draft. The criteria for inclusion of Delphi team members were as follows: having a Master’s degree, Ph.D. or MD in the mentioned fields of study with at least 5 yr of participation in relevant teaching and research. 

After purposive selection of the Delphi team members, the research team described the aim and scope of the research work for them. Then the first draft of the protocol was submitted to Delphi team members to be evaluated. The written comments were then assessed by the research team, most of included into the first draft of the protocol, resulting in the second draft. Afterward, the second draft of the protocol was submitted for evaluation through the second Delphi round. The mentioned process was repeated after new comments were gathered and applied.

Finally, after the third Delphi round, the Delphi team unanimously approved the face and content validity of the protocol contents and there were no more major comments and suggestions for correction and change. Therefore, the final version of the language intervention program was accessed at the end of the third Delphi round. 

## Results

The final language intervention protocol entitled “Interventional protocol on receptive vocabulary development of 12-48 months old cochlear implanted children” includes 5 interventional stages. The five stages are:

1- Drilling and Imitation; 2-Modeling; 3-Motor training; 4-Deliberate error correction; 5-Reinstatement and Generalization.

The main focus of the first and second stages in the present interventional package is on teaching nouns. When the child becomes equipped with almost 25-50 age-related nouns, the third stage will be started. This stage includes teaching of two-word phrases such as noun plus verb, noun plus adjective, and noun plus adverb, mainly based on motor training. The last two stages concentrate on improving the child’s ability to reinstate and generalize what he was taught in the previous stages by encouraging him to correct the therapist’s deliberate errors and to generalize his responses to different pictures and objects. 

This protocol needs to be conducted by a speech and language pathologist. However, parents or otherwise other care-givers are required to be presented at each therapeutic session and pay close attention and learn the intervention process, which they have to continue at home.

 Each stage consists of the description of the long-term aim and short-term objectives of this stage, the list of techniques utilized in the intervention process, the tools required for implementation of the intervention, the step by step detailed explanation of the intervention, how re-enforcement must take place and finally the indicators of success in this stage and permit to move forward to the next stage. 

The translated contents of the first stage, that is, “drilling and imitation” are illustrated in Table 1.

**Table 1:** The Drilling and Imitation stage in receptive vocabulary training

Stage 1: Drilling and Imitation Stage	
Long-term aim	1-Improvement of attention 2-Widening the range of receptive vocabulary (Nouns)
Short-term Objectives	Being able to imitate the therapist in pointing at the correct picture or object being named
Techniques	Repetition of the word in different pitches Pointing at the picture or object Reinforcement of the child upon successful imitation
Tools	Age-appropriate attractive pictures or objects
Intervention process	Ask the mother or another care-giver to watch carefully. Repeat the word and point at the relevant picture/object. Repeat 2-3 times. Help the child point at the relevant picture/object. Repeat the process for 3 more words in this manner and reinforce each correct imitated response. Continue with 2 more words if the child has not learned the intervention procedure until he learns. Ask the mother or another care-giver to continue the intervention process at home, with 20 selected words, similar to what she has seen the therapist does. At the next visit, evaluate the child’s performance Refer the child to other specialists (audiologists or pediatric neurologists) for further evaluations if the child does not meet the indicators of success criterion at this stage. Continue to the next intervention stage if the child meets the success criterion.
Reinforcement techniques	First reinforcement of every correct response with edible prizes (food) until 4 correct imitated responses are encountered. Then, reinforcement of every 2 correct responses (fixed rate schedule).
Indicators of Success	Receiving 80% correct imitated responses (that is, 16) from the 20 words already taught to the child

In order for therapists to be able to use age-appropriate words conveniently for every child, the present package contains several lists of content words (nouns, verbs, adjectives, adverbs) and functional words (only pronouns in this package) that normal hearing 12-48 months-old Persian speaking children understand, at different age levels (26, 31-34). Moreover, for each word, relevant pictures or drawings are prepared to illustrate it. Table 2 demonstrates age-appropriate nouns for the 12-18 months-old age group.

**Table 2:** Age-appropriate nouns for 12-18 months-old children

12-18 months	
Person	Father, Mother, Aunt, Uncle, Brother, Sister
Object	Ball, Car, Bed, Baby
Body(Limb)	Hand, Foot, Hair, Lip, Head
Food	Water, bread, milk, cookie
Animal	Fish, Cat, Dog, Mouse, Chicken

If the child does not meet the success criteria of any of the first 3 stages of training, the intervention will be discontinued at that stage and he or she will be referred to other specialists (audiologist, pediatric neurologists, pediatric psychiatrists, etc.) for further evaluation and treatment, and if not able to proceed with the intervention stages again, the child will be referred to receive special training suitable for children with learning disabilities. 

However, not being successful with the fourth and fifth stages, will lead the child to repeat an earlier stage as long as needed to accomplish the therapeutic objectives and to move forward.

A major characteristic of this protocol that is structured based on the normal process of vocabulary acquisition and language development in children, is its emphasis on a specific reinforcement system, based on behavioral psychologists’ point of view that noticeably emphasize on operant conditioning and step by step reinforcement of the child’s correct responses during training sessions. It works such that the reinforcement gradually moves from a very concrete prize (food) and frequent deliveries (for every one or two correct responses in a fixed rate manner) to an abstract prize (nodding of the head as a sign of approval) delivered randomly and unpredictably. This method is expected to dramatically increase the number of correct responses (35, 36). 

Furthermore, the performance of all 5 stages of this protocol is highly dependent on parents’ cooperation. The parents are asked to be present during each interventional session. This is mainly because of the importance of the parents’ behavioral reinforces like smiling, hugging or imitation of child’s correct responses. This seems to be one of the best means of communication and encouragement for language acquisition in language disordered children (35, 36). Moreover, the parents have an important role in continuation of the intervention process at home.

## Discussion

The main aim of this study was to develop a protocol for receptive vocabulary development of cochlear implanted children to help them acquire language in the limited golden time remaining for language acquisition. 

In 1996, the New York State Department of Health planned to develop and implement a language intervention program with the aim of enhancing and promoting language trainings quality, unity creation between language trainers, increasing parents’ collaboration and decreasing the training expenses. The target groups of this study were three-years-old or younger children with developmental delays, motor disorders, and communication disorders. In 2001, finally, the New York State Department of Health developed the final version of a guideline on this issue, with the help of pediatricians, speech and language pathologists, parents and other members of the rehabilitation group (11). 

Although this guideline mainly concentrates on early intervention, cognitive assessment and promoting rehabilitation quality, it is not a good choice for Persian speaking language impaired children, due to its cultural and linguistic differences. 

Another language intervention protocol which mainly focused on language impaired children and was specifically concerned with morphology and pragmatics was developed and conducted (10). In spite of various cultural and linguistic differences in different countries and societies, this is a structured protocol with specific action pictures that encourage the child to find and generalize the correct responses and has attracted the attention and interest of many speech therapists all over the world. However, it is not produced based on the normal process of language acquisition in children. 

Up to now, a number of language intervention protocols have been developed in Iran. One of the best ones is a guideline that was developed and carried out in 2012. Despite the positive aspects of this production, such as extension of the child’s vocabulary domain and perception of new words and phrases, it is not structured based on the normal process of vocabulary acquisition and language development in children. Moreover, the starting point for language training is not specified for the therapist, in terms of children in different chronological or developmental age groups (37).

Another language intervention protocol includes the 7 domains of pre-lingual disorders, developmental language disorders, hearing impairments, speech sound disorders, dysphagia, stuttering, and dysarthria (38). This recently-produced protocol is a rather comprehensive diagnostic and treatment protocol for early intervention in children who suffer from various aspects of speech and language impairments. Thus, unlike the present protocol, it is not mainly concerned with receptive vocabulary development of cochlear implanted children.

The insufficiencies and weak points, as well as different target groups of the different existing language intervention protocols, and also the need to have a Persian culturally-adapted protocol specifically designed for promotion of receptive vocabulary in Iranian cochlear-implanted children, encouraged the authors to design a language intervention protocol with the previously described characteristics, in order to guide speech therapists and to prevent their confusion in training cochlear implanted children. 

One of the limitations of this study is that the authors did not adapt the words used in the different training stages of the protocol, with that of different Iranian dialectical and cultural differences. Therefore, other researchers do so. Other researchers must start to provide other educational packages relevant to other aspects of language development, including pragmatics and functional words such as adverbs and prepositions.

**In conclusion,** although the intervention process, as well as the words used in this protocol, are based on the process of normal language development in 12-48 months-old Persian speaking children, they can be adapted in accordance with the developmental norms and linguistic features of any other language. 

## Author’s contribution

Monshizadeh L proposed the main concept and idea of the research, performed the research and wrote the paper,

 Vameghi R made critical contribution to the concept and design of the research and performed critical revision related to content of the manuscript, Sajedi F, Yadegary F, Rahimi M and Hashemi SB contributed equally in the concept and design of the study. 

All authors agreed to be accountable for all aspects of the work in ensuring that questions related to the accuracy or integrity of any part of the work are appropriately investigated and resolved.
